# Twitter use at the 2016 Conference on the Science of Dissemination and Implementation in Health: analyzing #DIScience16

**DOI:** 10.1186/s13012-018-0723-z

**Published:** 2018-02-20

**Authors:** Caitlin G. Allen, Brittany Andersen, David A. Chambers, Jacob Groshek, Megan C. Roberts

**Affiliations:** 1 Rollins School of Public Health, Emory University, 1518 Clifton Rd, Atlanta, GA 30322 USA; 2 Division of Emerging Media Studies, Boston University, 704 Commonwealth Avenue, Boston, MA 02215 USA; 3 Division of Cancer Control and Population Sciences, National Cancer Institute, 9609 Medical Center Drive, Rockville, MD 20850 USA

**Keywords:** Social media, Dissemination, Implementation science, Health, Data analytics

## Abstract

**Background:**

Poor dissemination of research findings may hamper the reach and impact of scientific discoveries. One key emerging platform for research dissemination is social media, including Twitter. While Twitter and other social media are increasingly being used to disseminate research content presented during scientific conferences, few studies have investigated the extent to which these tools are used throughout conferences and how they are being used. The aim for this study was to better understand the use of Twitter during the 2016 Annual Conference on the Science of Dissemination and Implementation in Health (D&I conference).

**Methods:**

We performed an analysis of Twitter use before, during, and after the 2016 D&I conference, which took place from December 14 to 15. All tweets (posted between December 1 and 31) that included the conference-specific hashtag (#DIScience16) were assessed. We identified 2639 tweets using the data analytics platform NUVI. We used NUVI software to generate statistics about reach, influence, mentions, and origin of the tweets. Individual tweet content was also assessed using DiscoverText and coded for disease category, implementation outcomes discussed, category of tweet, and conference track.

**Results:**

A total of 2639 tweets were analyzed; 89.1% of the tweets were posted during the conference. A total of 389 unique users participated on Twitter, representing 31 states and 22 locations outside of the USA. Most (56.8%) tweets were re-tweets and were used for scientific promotion (50.6%). Key conference speakers and implementation outcomes (de-implementation, adaptation, and fidelity) were commonly discussed.

**Conclusions:**

Our findings reveal that Twitter was used as a platform during the D&I conference, both to facilitate conference discussion and to promote scientific ideas. This work contributes to the existing data analytics and implementation science literature in two major ways: (1) by advancing knowledge of how social media is used during annual academic conferences and (2) by providing a deeper understanding of themes and emerging areas of interest in the dissemination and implementation sciences. Knowing specific topics of interest can help planners and scientists better understand the landscape of current and future implementation research and encourage new research dissemination strategies.

## Background

The dissemination of research findings can help propel important areas of research forward and promotes communication of science [1–5]. Although dissemination of findings is a priority among researchers and funders alike [2, 6], studies have demonstrated suboptimal dissemination efforts, with a recent study reporting that 73% of researchers spend less than 10% of their time on dissemination activities [7]. While researchers’ participation in the active dissemination of their work varies and can take many forms (e.g., press, policy briefs), social media provides a new communication channel for researchers to actively reach the public, other scientists, and stakeholders [1, 8].

Social media outlets provide a platform to participate in scientific discussions and disseminate research findings. Twitter, specifically, is a user-friendly application that can be accessed via mobile devices and computers [9]. Twitter allows users to target delivery of information and can complement traditional research dissemination methods. This application is especially notable with the dissemination of public health information [10–16]. The Twitter platform has been a growing outlet for key public health agencies, organizations, and journals including the Centers for Disease Control and Prevention (844,500 followers), JAMA (216,000 followers), and National Cancer Institute (133,000 followers). Despite growing evidence about the importance of social media in disseminating research findings and rising use of social media among key research organizations, there is limited information about researchers’ attitude, perceptions, and use of social media, including Twitter [17].

Today, in an effort to promote engagement and support dissemination of research findings, planners of scientific conferences often create a unique hashtag for annual meetings to encourage such discussion and promotion [18–21]. The use of hashtags (#) on Twitter allows followers to collate discussions around specific topics, including scientific themes or events. Previous studies have demonstrated that Twitter is used before, during, and after medical and other scientific conferences as an extension of the scientific discourse [22–25]. These studies have demonstrated that use of social media at these conferences enhances the overall impact of a conference by engaging attendees and virtual followers, in addition to facilitating dialog and professional networks beyond the conference location and attendance [26].

The present study focused on applying existing network analysis methods to assess the content of the Annual Conference on the Science of Dissemination and Implementation in Health (D&I conference), where scientists gather to discuss trends in the field of dissemination and implementation science [27]. This conference is hosted by the National Institutes of Health [28] and AcademyHealth [29] to promote discussion and dissemination of advances in the field. A large component of the conference is dedicated to disseminating research findings, and social media is one way to extend the reach of the conference beyond the walls of the conference hall. The purpose of this analysis was to determine the usage and impact of the social media platform Twitter during the 2016 D&I conference, which used an official hashtag: #DIScience16. The aims of this paper are (1) to advance the knowledge of how social media was used throughout the annual D&I conference using the official conference hashtag and (2) to provide insight into key themes and emerging areas of interest in the field of dissemination and implementation science.

## Methods

We performed a retrospective analysis of Twitter use during the 9th annual D&I conference held in Washington D.C. Tweets and their associated metadata were collected using NUVI, a social media mining tool [30]. We collected tweets that used the official conference hashtag (#DIScience16) between December 1 and 31, 2016 (conference dates were December 14–15, 2016). Preliminary sensitivity analysis of related hashtags (e.g., #DIScience) were conducted. Due to the limited use of non-conference hashtags and active promotion of the official conference hashtag throughout the conference, we focused on the analysis of #DIScience. A total of 2639 Tweets were identified.

For each tweet, NUVI provided data about the date and time of the tweet, ReShare (e.g., whether the tweet has a parent post [re-tweet] or whether the tweet does not have a parent post [original]), reach (e.g., potential number of people who may see the post), Klout score [31] (e.g., influence score, users reach and engagement), and state and country of the Twitter account. In addition, we developed a coding scheme to identify whether a hyperlink was used in the tweet, the number of mentions (use of @ symbol), whether speaker(s) were mentioned, and mention of a disease group. This coding scheme also included category of tweet, conference track, and mention of implementation outcomes [32]. An initial codebook was developed using an iterative process, beginning with a priori codes and adding additional code response categories using a consensus process as themes emerged from the data. Two coders (CGA and MCR) pilot tested the codes on 30 randomly selected tweets. The coders then met for consensus and updated the codebook based on discrepancies. After the initial consensus, 30 additional tweets were coded and a final codebook was adopted (Table 1).

**Table 1:** Coding scheme and examples of tweets

Category of tweet	
Code and definition	Example
Social: social aspects of conference, promotion of social events, conversations between users	#DIscience16 We’ll be there w/ @HugginsJoe Recently started podcast https://t.co/LtwgAUtCLB would love to meet w/ others who are podcasting https://t.co/usGCI9nYnk
Logistics: room changes, where to meet for sessions	Come discuss #deimplementation with Dr. Wynne Norton at lunch today! #DIscience16 https://t.co/JW7g77NjIZ
Conference promotion: general promotion about conference, events surrounding conference	@AcademyHealth outstanding opening keynote by #RoyRosin at #DIscience16. Great start to a conference that has grown by leaps&bounds in 9 yrs’ CURATION: Science of Dissemination &amp; Implementation in Health-NIH/AcadHealth DEC 14–15 #DIScience16
Scientific promotion: focus on area of research that should be pursued	‘We can’t manage what we cannot measure.’ - @LJDamschroder #DIScience16’ Submit protocol papers to Implementation Science as early as possible. Timing requirements are changing for 2017. #DIscience16’ How we advance science is thinking theoretically &amp; testing theories. We’re not seeing enough in #impsci. - @AnneSales4 #discience16.
Self-promotion: mention of individual’s research, interests, ideas	Looking fwd to sharing my research at the #DIscience16 conference! See u next week? Adaptation studies involving VA QUERI. Diffusion of excellence initiative, incl advance care planning group visit model. #DIscience16
Conference track	
Code and definition	Example
Behavioral health	CRTKL’s Samira Pasha will present work on #space #design for #behavioralhealth at #DIscience16. @NIH @AcademyHealth https://t.co/zuDy6wPzO6 https://t.co/eobpePujCz
Big data and technology for dissemination and implementation	Impact of social media &amp; tech innovation on work we do How can we level playing field so innovations do not go out of date? #DIscience16 https://t.co/dbY7dTK72f
Clinical care settings	Headed to #DIscience16 tomorrow? CHOIR investigators will be presenting work on #PatientCentered care, #telehealth, and more. See you there!
Global dissemination and implementation	Attending #DIscience16? Come join me in Virginia A today at 12:15p Insights from Salzburg Global Seminar #SGShealth https://t.co/cdAaEeCkyo https://t.co/PfwIDPFUWN
Models, measures, and methods	.@Greg_Aarons kicking off a great panel on fidelity and adaptation. #DIscience16 https://t.co/n3mFIN21AM
Prevention and public health	How can dissemination &amp; implementation science optimize #health? #DIscience16 @NCI_ImplSci #DIscience16 Would be interested in meeting with others in #SuicidePrevention https://t.co/3w1DJQmETZ
Health policy D&I	Hey #DIscience16 folks, check out #All4Evidence @AcademyHealth for great convo on health policy &amp; evidence. 1/2 Be sure to check out PolicyLab’s Jordan Price at the @AcademyHealth @NIH #DIscience16 Conf. today at 5:30 pm! https://t.co/Kbc0mrYjtu https://t.co/924QKBMZlT
Plenary sessions	Kicking off #DIscience16 feat. @AcademyHealth’s @DrSimpsonHSR &amp; @NCIDAChambers! Over 1000
Poster sessions	Ce-PIM researchers will be presenting posters at @AcademyHealth &amp; @NIH D&amp;I Conference 12/14! Stop by and chat with us! #DIScience16 #impsci https://t.co/qD7uJRRJcH
Precision medicine	Implementation science can increase the efficiency of moving #PrecisionMedicine from trial to practice so says Brian Mittman #DIscience16 #genetics research at #DIscience16 https://t.co/U8yqaA35h1
Promoting health equity and eliminating disparities	Positive deviance methods w transparent rpting to reduce disparities in Cleveland. Hurray! #DIscience16
Targets of implementation research*	
● Implementation outcomes: feasibility, fidelity, penetration, acceptability, sustainability, uptake, cost ● Service outcomes: efficacy, safety, effectiveness, equity, patient centered, timeliness ● Client outcomes: satisfaction, function, symptomology ● Added by research team: de-implementation, facilitation, organizational readiness, adaptation	

*Adapted from Proctor E. et al. [32]

The two coders then independently coded 300 tweets. Upon completion of manual coding, we imported data into DiscoverText (Amherts, 2017), a software used for human- and machine-learning coding [9]. We used the machine learning feature of DiscoverText to code the remaining tweets. The results of the machine-learning coding were reviewed by the three coders, and a cutoff score of 0.5 was established to allow for classification of tweets into the specific categories. Tweets were also checked by hand for keywords (e.g., genomics, equity) to ensure the quality of coding.

After data abstraction, descriptive analyses were conducted using SPSS version 24. We used basic descriptive statistics and bivariate analyses to compare content of tweets (1) before, during, and after the conference, as well as (2) original versus re-tweets. Words with their frequency of use were pulled from DiscoverText data to generate a WordCloud representing the most frequently used terms in the Twitter data [9].

## Results

We identified 2639 Tweets that included the official conference hashtag. There were 389 unique users, which reflected 35.3% of the total number of conference participants (*n* = 1103). A total of 22 countries were represented with 63% of tweets coming from users in the USA (*n* = 1662). Thirty-one states were represented. Other countries included Canada (*n* = 72) and the UK (*n* = 69). Of those users in the USA, the most common states/district were North Carolina (31.0%), Washington D.C. (17.4%), and Indiana (5.0%). The majority of tweets occurred during the conference (89.1%) (Table 2).

**Table 2:** Descriptive information about tweets (*N* = 2639)

Category	*N* (%)
Re-share	
Yes	1499 (56.8)
Timing	
Before conference	99 (3.8)
During conference	2352 (89.1)
After conference	188 (7.1)
Category of tweet (*N* = 2509)	
Scientific promotion	1269 (50.6)
Conference promotion	751 (29.9)
Self-promotion	265 (10.6)
Social	99 (3.9)
Logistics	73(2.8)
Other	52 (2.0)
Conference tracks (*N*=2444)	
Models, measures, and methods	669 (27.4)
None	602 (24.6)
Plenary	341 (14.0)
Big data and technology for D&I research	148 (6.1)
Behavioral health	141 (6.1)
Poster	107 (4.4)
Global D&I	97 (4.0)
Prevention and public health	84 (3.4)
Clinical care settings	75 (3.9)
Health policy D&I	70 (2.9)
Health equity	60 (2.5)
Precision medicine	49 (2.0)
Disease mention	
Yes	64 (2.4)
Implementation word used	
Yes	473 (17.9)
Mentions (@)	
Yes	1063 (40.3)
Number of mentions	
0	1575 (59.7)
1	750 (28.4)
2	206 (7.8)
3+	135 (5.1)

### Influence and reach

Klout scores (users’ influence measured by reach and engagement) ranged from 10 to 82 with the average score being 42.0 (SD = 9.9). The top three influencers were (1) *AHRQNews*, which is an account hosted by US Department of Health and Human Services and the Agency for Healthcare Research and Quality (co-sponsors of the conference), which contributed five unique tweets; (2) *drnic*, which contributed one tweet and is a personal account by the CEO of Stark & Wayne; and (3) *7wData*, an account from Belgium that focuses on business, information management, analytics, and digital transformation that contributed one tweet. Reach scores (calculated by the total number of users’ followers) ranged from 4 to 95,436 followers (CDC’s Division of Cancer Prevention and Control).

The most frequent users were *ImpSciX* (257 tweets), *RMIRECC* (239 tweets), and *ConsortiumforIS* (211 tweets). In sum, these three users accounted for 707 tweets (26.8%) of all conference-related tweets. *ImpSciX* is associated with ImpSci Exchange a “one stop shop resource for implementation science, curated by UNC’s Translational and Clinical Sciences Institute” (https://impsci.tracs.unc.edu). *RMIRECC* is the RockyMountain Mental Illness Research, Education and Clinical Centers for veterans’ suicide prevention (https://www.mirecc.va.gov/visn19/), and *ConsortiumforIS* is “a joint endeavor of RTI International and UNC School of Global Public Health” (http://consortiumforis.org/). Two of these three users were located in North Carolina.

### Use of social media during the conference

Over half of the tweets from the conference time period were re-shared (56.8%). Among re-shared tweets (compared to original tweets), similar trends were seen in timing, category, disease mention, implementation outcomes, and number of mentions. The most frequent re-tweets discussed scientific ideas and quotes from conference presenters (Table 3). The most re-tweeted content was about developing interventions that account for context and/or described fidelity and adaptation, which were common scientific themes throughout the conference. Most of the tweets mentioned an individual presenter/researcher either by tagging them or by name. In addition, most of the re-tweets included a link.

**Table 3:** Top retweets and content

Content	Number of tweets
Stop dev interventions as if context is separate. Instead, develop interventions IN context=Fidelity+adapt. #DIscience16 @SusanMichie	33
Asking the big picture questions of quality improvement, research, and evaluation #DIScience16 #SGSHealth #publichealth https://t.co/8Xj8yHhUzY	19
Roy Rosin: if u r failing the right way, u aren’t failing. U r testing hypotheses #DIscience16	17
RT @taren_swindle: “RCTs are the least adaptive mechanism for learning I know.” - Goldman #DIscience16	14
Barrier to evidence-based de-implementation: Rare publication of null findings. #DIScience16	13
Roy Rosin bringing rapid testing concepts to #DIscience16--thinking way outside of the typical research box!	13
.@PennMedNews: Roy Rosin: Failing the right way? An innovation #ImpSci culture fails fast,cheaply .@AcademyHealth #DIscience16 #healthcare	12
So true! Just giving folks a checklist is NOT enough! Why do we keep making the same mistakes?? #DIScience16 https://t.co/s5f2Yh6oHa	12
Pragmatic = actionable, sensitive to change, low burden, important to stakeholders - C. Stanick #DIscience16 https://t.co/BkOJtoJhdF	11
‘It’s amazing how often we solve the wrong problem’ R. Rosin #DIScience16 #impsci #mtdirc	11
#DIscience16 @SusanMichie 3 issues related to fidelity https://t.co/Orsgz40eUp	10
#DIscience16 @SusanMichie Problem of poor Fidelity https://t.co/P9VaWanV5W	10
#DIscience16 great story from Roy Rosin about the importance of observing what people do rather than relying on what people say.	10
If you don’t, you can’t understand what’s going on which is a prerequisite for improvement... #DIscience16 https://t.co/NamKUf0Rt9	10
Just because something is novel does not mean it is better. Hilda Bastian @nlm_news #DIScience16 https://t.co/dWxWZoSvzj	10
Loving the #design methodology emphasis of Roy Rosin plenary at #DIscience16. Hope to see more #designthinking through the whole conf!	10

The majority of tweets occurred during the 2-day conference. Over the course of the conference, Twitter activity peaked between 1:00 and 1:59 PM on December 14 (day 1), with 292 tweets per hour. The second most active time was between 12:00 and 12:59 PM on December 14, with 228 tweets per hour. This increased activity aligned with the first scientific sessions of the conference that took place between 12:15 and 1:45 PM. The majority of tweets that occurred between 12:00 and 1:59 PM on day 1 were re-tweets (66.9%), indicating that users actively shared and recirculated ideas from the conference.

### Twitter discussion of key scientific areas in implementation science

The majority (50.6%) of tweets that we analyzed included scientific promotion and conference promotion (29.9%). The primary conference track discussed was “models, methods, and frameworks” (27.4%) (Tables 1 and 2).

We also assessed whether there was a disease mention or mention of implementation science-related themes. Only 2.4% of tweets explicitly mentioned a disease. Of those that mentioned disease, 17.2% were cancer and chronic pain (12.9%). 17.9% of all tweets included some reference to implementation methods or outcomes. The most common implementation themes within this category were adaptation (24.5%), de-implementation (16.9%), and a combination of acceptability and feasibility (14.8%). Among tweets that included implementation themes and outcomes, 87.5% were during the conference, and of those tweets that included a disease mention, 89.1% were during the conference.

When comparing the Twitter content across time, the majority of tweets before and after the conference were about conference promotion (29.6, 48.0% respectively); however, during the conference, the majority of the tweets were about scientific promotion (54.2%). The conference track also shifted before, during, and after the conference. Before and after the conference, the majority of users did not discuss a conference track (63.1, 37.0% respectively); however, during the conference, most tweets were about the models, methods, and frameworks track (39.6%) (Table 4).

**Table 4:** Variation in content before, during, and after the conference

	Before *N* (%)	During *N* (%)	After *N* (%)
Category (*N* = 2509)			
Conference promotion	29 (29.6)	637 (28.5)	85 (48.0)
Logistic	8 (8.2)	62 (2.8)	3 (1.7)
Other	2 (2.0)	47 (2.1)	3 (1.7)
Scientific promotion	7 (7.1)	1210 (54.2)	52 (29.4)
Self promotion	35 (35.7)	208 (9.3)	22 (12.4)
Social	17 (17.3)	70 (3.1)	12 (6.8)
Total	98 (100)	2234 (100)	177 (100)
Conference track (*N* = 2444)			
Behavioral health	1 (0.9)	120 (5.5)	20 (11.6)
Big data D&I	0 (0)	132 (6.1)	16 (9.2)
Clinical care setting	3 (2.9)	70 (3.2)	2 (1.2)
Global D&I	1 (0.9)	85 (3.9)	11 (6.3)
Models, measures, and methods	2 (1.9)	641 (39.6)	26 (15.0)
None	65 (63.1)	473 (21.8)	64 (37.0)
Plenary	9 (8.7)	323 (14.8)	9 (5.2)
Poster	10 (9.7)	88 (4.1)	9 (5.2)
Prevention and PH	7 (6.7)	72 (3.3)	5 (2.9)
Health policy and D&I	1 (0.9)	68 (3.1)	1 (0.6)
Health equity	3 (2.9)	55 (2.6)	2 (1.2)
Precision medicine	0 (0)	41 (1.9)	8 (4.6)
Total	103 (100)	2168 (100)	173 (100)

**Fig. 1:**
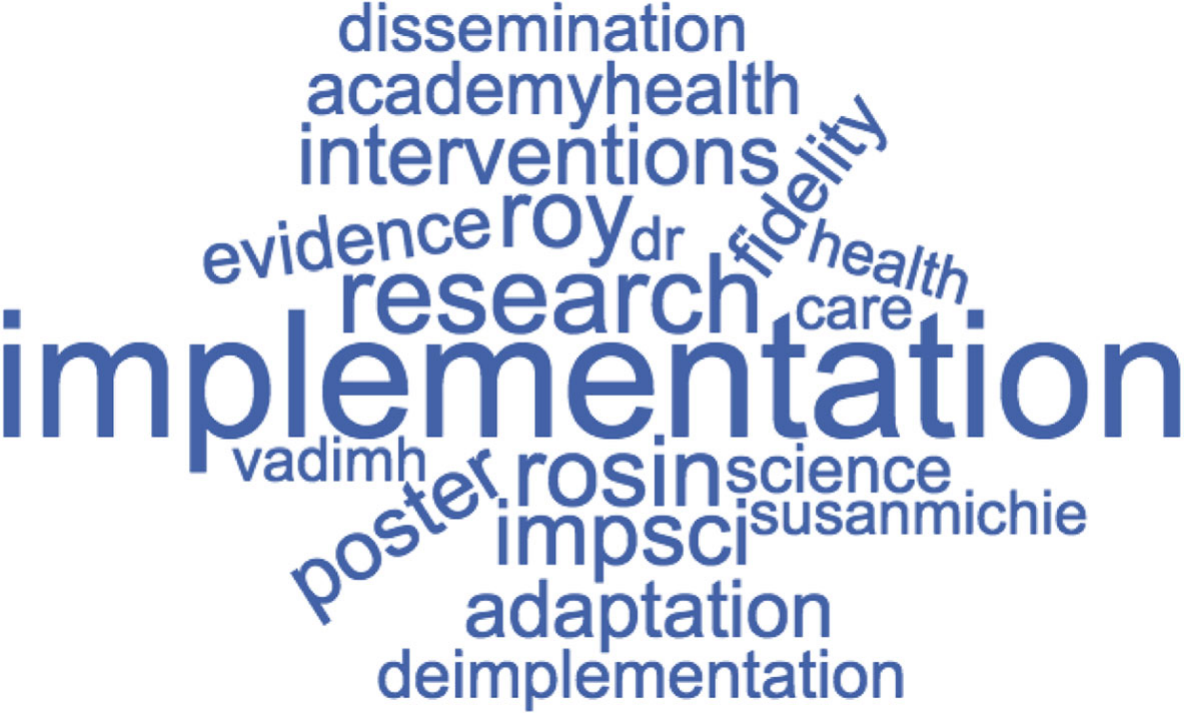
WordCloud and Word Frequencies. Roy Rosin (319), implementation (286), research (180), impsci (145), interventions (141), poster (141), adaptation (129), academyhealth (118), fidelity (131), evidence (112), deimplementation (107), dissemination (109), science (111), susanmichie (103)

Our team also analyzed the most commonly used words in the dataset by generating a word cloud from the DiscoverText data (Fig. 1). Results from the WordCloud visually depict the frequencies of words used in all Twitter content. Implementation was the most commonly used word and was used in 10.8% of the tweets followed by research (6.8%), Roy Rosin (12.1%) (combined use of Roy and/or Rosin a plenary speaker), and impsci (5.5%).

## Discussion

The annual D&I conference is the pre-eminent conference for the dissemination and implementation research community. Our study is the first to describe how individuals engage with this conference through social media, which offers insight for scientists and conference planners alike. We examined demographics, popular content, and trends in the data, highlighting the reach and influence of tweets. Meaningful discussions and promotion of relevant research topics were extended through the use of Twitter during and after the conference. Our results advance the knowledge of how social media is used during academic conferences and offer a deeper understanding of key themes and emerging areas of interest in dissemination and implementation science.

### Advancing knowledge of social media use during academic conferences

Twitter is an emerging space for researchers to communicate and share findings [1, 33]. Over one third of conference tweeters were engaged in Twitter throughout the month of December, and 2352 tweets occurred over the course of the 2-day conference. These findings demonstrate substantive engagement via Twitter, particularly concentrated during the conference dates, which aligns with evidence that Twitter is most commonly used to communicate about live events [34]. The total number of tweets surrounding the D&I conference was substantially greater than the use reported in other conference settings [19, 26, 35], likely reflective of the clear messaging from conference planners about the specific conference hashtag and promotion of social media during the abstract submission process, leading up to, and throughout, the conference.

In addition, conference attendees are likely dissemination and implementation scholars and consequently have bought into the value of strategic dissemination of research evidence. Other studies have demonstrated the poor use of social media among scholars; however, D&I conference attendees may be unique in their level of social media activity, setting the pace for other researchers who seek to disseminate their findings in targeted, relevant ways. Future research could further explore the characteristics of dissemination and implementation researchers to see if they align with findings from other studies that have examined correlates of higher social media use, such as institutional support for dissemination via social media and research topics that may be more relevant for dissemination to the public [7, 17].

Although a large majority of tweets came from accounts in the USA, 21 other countries were represented among Twitter users discussing the conference. These findings demonstrate that scholars have an interest in engaging in scientific discourse related to conferences, even if they are unable to physically attend. This improves the visibility of the conference content and of the conference attendee messages.

In addition, these findings advance our knowledge of how social media is used during academic conferences. The most active times for Twitter were during conference sessions, allowing attendees to virtually attend other sessions by receiving key messages from concurrent sessions through Twitter. This unique feature of Twitter promotes ongoing discussion of topics both for people attending virtually and in person, a key goal identified by D&I conference planners [27]. This information demonstrates the potential opportunity to expand reach and engagement in national scientific conferences.

Future conference planners can continue to support research dissemination via Twitter by including the Twitter handle as part of abstract submission, encouraging presenters to include a Twitter handle in their presentations, and creating an active social media environment by creating an easy-to-remember conference hashtag and actively tweeting throughout the conference. These strategies can also promote the communication of conference-related research findings to a wider audience.

### Emerging areas of dissemination and implementation research

Our findings also provide a deeper understanding of key themes and emerging areas of interest in dissemination and implementation research. Not surprisingly, half of all tweets were scientific promotion, with a specific focus on sharing implementation models, methods, and frameworks during the conference. This focus is especially important given that current literature often lacks implementation frameworks and rigorous implementation science methods [36, 37].

Future research could leverage the D&I conference platform to identify ways to incorporate social media strategies in dissemination efforts in order to help expand relevant research findings to academic and non-academic audiences. Such efforts could help improve the speed of delivery of research findings by promoting commentary among Twitter users, exploring the use of Twitter as an educational tool for dissemination and implementation researchers, and considering opportunities for improving relevance of findings to non-academic audiences.

Key themes identified by the conference planning committee include de-implementation, creative ways to implement (i.e., keynote speaker, Roy Rosin’s topic, “The Implementation Conundrum”), and the tension between adaptation and fidelity. The most common implementation themes (adaptation, de-implementation, and acceptability and feasibility) reflect the goals of conference planners. The most frequently re-shared tweets also reflected these themes and commonly mentioned fidelity, adaptation, and the keynote speaker, Roy Rosin [27]. Knowing the impact of keynote speakers on driving the conference dialog can help planners and scientists plan and promote discussion around urgent research areas, conference activities, future efforts, and conference themes moving forward.

### Limitations

While this is the first report to describe how social media is used during an implementation science conference, it has several limitations. First, this paper provides cross-sectional snapshots of content from the 2016 D&I conference. While we were able to document trends before, during, and after the conference, we did not examine annual trends. Thus, this analysis serves as a baseline of conversations that took place surrounding the 2016 meeting and should be considered a snapshot in time. Follow-up studies would need to be conducted to understand changes in Twitter use and content over several years. In addition, we only captured tweets that included the official hashtag of the conference; however, it is possible that conference-related tweets were sent without the official hashtag. We did not independently explore other common, related hashtags (e.g., #impsci, #implementation); however, our analysis demonstrated that these were commonly used in parallel with the official tag. Future studies may include a broader search criteria and extend to assess use of other social media platforms such as Facebook.

## Conclusions

This report offers a unique perspective about the field of dissemination and implementation science and an opportunity to advance the dissemination of research findings across settings. Our findings support the conference’s unique mission to disseminate research findings among scientists and a wider audience. In the future, researchers should continue to observe trends in Twitter use surrounding the D&I conference and compare trends across time to better understand the landscape and trajectory of the D&I research field. Conference planners can continue to promote the use of Twitter to help spread the conversation beyond the conference and to the public and non-attending researchers. This study offers support for the use of Twitter at scientific conferences, as it provides a unique outlet to promote both conference activities and emerging research trends in implementation and dissemination science.
